# Baclofen in the Treatment of Patients With Alcohol Use Disorder and Other Mental Health Disorders

**DOI:** 10.3389/fpsyt.2018.00464

**Published:** 2018-09-28

**Authors:** Roberta Agabio, Lorenzo Leggio

**Affiliations:** ^1^Section of Neuroscience and Clinical Pharmacology, Department of Biomedical Sciences, University of Cagliari, Monserrato, Italy; ^2^Section on Clinical Psychoneuroendocrinology and Neuropsychopharmacology, National Institute on Alcohol Abuse and Alcoholism Division of Intramural Clinical and Basic Research and National Institute on Drug Abuse Intramural Research Program, National Institutes of Health, Bethesda, MD, United States; ^3^Medication Development Program, National Institute on Drug Abuse Intramural Research Program, National Institutes of Health, Baltimore, MD, United States; ^4^Center for Alcohol and Addiction Studies, Brown University, Providence, RI, United States

**Keywords:** GABA_B_, baclofen, alcohol use disorder, mental health disorders, anxiety, mood disorders

## Abstract

A limited number of medications are approved to treat Alcohol Use Disorder (AUD). Furthermore, the magnitude of their therapeutic effect is relatively modest, suggesting the potential for subtypes of patients who respond to a specific medication. The use of these medications is also limited in clinical practice by a series of contraindications such as medical comorbidities and/or concurrent use of other medications. In recent years, animal and human studies have been conducted to evaluate the efficacy of baclofen, a GABA_B_ receptor agonist approved for clinical use as a muscle relaxant, in the treatment of AUD. However, these studies have yielded contrasting results. Despite this discrepancy, baclofen is often used off-label to treat AUD, especially in some European countries and Australia. Recently, several factors have been considered to try to shed light on the potential reasons and mechanisms underlying the inconsistent results obtained until now. The presence of a psychiatric comorbidity may be amongst the abovementioned factors playing a role in explaining different responses to baclofen treatment in terms of alcohol drinking outcomes. Therefore, the aim here was to conduct a narrative review of the scientific literature related to the use of baclofen in AUD, both in patients with and without concomitant psychiatric disorders. All clinical studies (randomized and controlled, open-label, retrospective, human laboratory studies, and case reports) were analyzed and discussed, bearing in mind other potential factors that may have influenced baclofen response, including dose administered, severity of AUD, use of other psychosocial therapies, and the presence of physical disorders. This review indicates that the most frequent psychiatric comorbidities in patients affected by AUD undergoing baclofen treatment are anxiety and mood disorders. Unfortunately, no definitive conclusions can be drawn due to the lack of specific analyses on whether baclofen efficacy is different in AUD patients with comorbid psychiatric disorders vs. those without. Therefore, it will be critical that psychiatric comorbidities are considered in the planning of future studies and in the analysis of the data, with the ultimate goal of understanding whether subtypes of AUD patients may respond best to baclofen.

## Introduction

### AUD and need for other medications

Alcohol Use Disorder (AUD) is a severe and complex mental disorder mainly characterized by excessive alcohol consumption and the inability to control it (1). Despite being one of the leading causes of morbidity and mortality worldwide (2, 3), only a limited number of medications are available to help AUD patients achieve abstinence or reduce their alcohol consumption (4, 5). In most countries (e.g., Europe, North America, Australia, parts of Asia, and Africa), these medications include disulfiram, naltrexone, and/or acamprosate (6, 7). Recently, nalmefene was also approved in Europe (6). Unfortunately, only a very small number of AUD patients receive these medications. In the US, <10% of people with AUD receive any type of alcohol treatment (8) and fewer than 4% receive a medication as an intervention or treatment (9).

Possible reasons for the underutilization of medications for AUD include a lack of knowledge, by both patients and physicians, and the misperceptions that medications do not work or that AUD should not be treated with a medication (6). However, certain characteristics of AUD medications also contribute to discouraging physicians from prescribing them. Globally, the magnitude of the therapeutic effects of AUD medications is relatively modest (4), and patient response may differ to a specific drug (10, 11). AUD medications are also contraindicated in patients with some medical comorbidities (4, 5, 12, 13). For instance, naltrexone and disulfiram are contraindicated amongst patients with clinically-relevant liver diseases; on the other hand, acamprosate is contraindicated in patients with kidney failure. Furthermore, disulfiram should not be used in patients who are not capable of understanding the risks of consuming alcohol while they are taking disulfiram. In clinical practice, these conditions are frequent and contribute to a further reduction in the available treatment choices for AUD patients. Another frequent condition in clinical practice is the presence of comorbid mental disorders such as substance use disorder, mood disorder, and/or anxiety disorder, especially among patients with more severe AUD (14). The use of medications for AUD among these latter patients is even lower than among AUD patients without other comorbid mental disorders, because of the concern of medication-alcohol interactions (15). For all these reasons, the identification of new, safe, and effective medications is a critical priority in the field of AUD treatment (16–18).

### Baclofen and AUD

Baclofen is a GABA_B_ receptor agonist approved for clinical use as a muscle relaxant. In recent years, animal and human studies conducted to evaluate the efficacy of baclofen in the treatment of AUD have yielded contrasting results (19). Despite this discrepancy, baclofen is often used to treat AUD, especially in European countries and Australia, as a consequence of the wide off-label prescription of the drug by general practitioners (20). Recently, several factors have been considered and analyzed to shed light on the potential reasons and mechanisms underlying the inconsistent treatment results obtained until now. It has been suggested that the presence of a psychiatric comorbidity may be amongst the abovementioned factors playing a role in explaining different responses to baclofen treatment, in terms of alcohol drinking outcomes. Preclinical and clinical findings clearly indicate a key role of the GABA_B_ receptor in depression and anxiety disorders (21, 22). Some studies found that baclofen reduces anxiety levels in AUD patients (23–25). Conversely, other studies did not find significant effects of baclofen on anxiety levels in AUD patients (26), or they found that patients without comorbid mental disorders achieved better results compared to patients with comorbid mental disorders (27–29). Therefore, the aim of the present paper was to conduct a narrative review of the scientific literature related to the clinical use of baclofen in AUD to achieve abstinence or reduce alcohol consumption, both in patients with and without concomitant psychiatric disorders. All clinical studies (randomized and controlled, open-label, retrospective, test-tube lab research, and case reports) were summarized and discussed, bearing in mind other potential factors that may have influenced baclofen response, e.g., baclofen dose, severity of AUD, use of other psychosocial therapies, and the presence of medical comorbidities.

### Methodology

Data were obtained for the narrative mini-review by searching the published medical literature in Medline (PubMed) up to May 2018. There were no language restrictions; the search was limited to humans. The search terms used included alcoholism or AUD or alcohol dependence AND baclofen.

## Review of the scientific literature related to the use of baclofen in AUD both in patients with and without concomitant psychiatric disorders

### Case reports

A few case reports describe the results obtained by the use of baclofen in AUD patients [see Table 1; (30–35)]. All these patients suffered from severe AUD, as shown by their high level of alcohol consumption at baseline, the lack of a response to other previous treatments for AUD, and the presence of one or more comorbid psychiatric disorders, such as anxiety, mood disorder, schizophrenia, and bulimia. Patients received daily doses of baclofen, ranging from 50 to 120 mg, and were followed for a timeframe ranging from 8 to 52 weeks. In all these patients, baclofen administration led to alcohol abstinence or to a marked reduction in alcohol consumption. On the other hand, possible effects of baclofen on the severity of the other mental disorders were not reported by most of the studies and, when reported, the results were contrasting.

**Table 1:** Case reports.

	**Participants' characteristics**			**Baclofen treatment**		**Clinical effects of baclofen treatment**					
**References**	**Gender**	**Comorbidity**	**Baseline DDD**	**Mean daily dose**	**Weeks**	**Final DDD**	**Anxiety**	**Depression**	**Schizophrenia**	**Bulimia**	**Stuttering**
(30)	1 M	Schizophrenia Anxiety	~16	75 mg	24	0	↔	–	↓	–	–
(31)	1 M	Anxiety	~20	120 mg	36	0	↓	–	–	–	–
(32)	1 M	Stuttering Depression	~16–24	90 mg	>52	2	-	↓	–	–	↓
(33)	1 M	Anxiety Depression	~5–12	100 mg	40	2–3	↔	↔	–	–	–
(34)	3 M; 1 F	Depression Bipolar disorder	~7–30	50-125 mg	20–36	0	–	–	–	–	–
(35)	1 F	Anxiety Bulimia	~20	120 mg	8	0	–	–	–	↔	–

*DDD, Drinks per Drinking Day (1 drink = ~12 g of pure alcohol); F, Female; M, Male*.

### Retrospective studies

A series of retrospective studies evaluated the efficacy of baclofen among large samples of AUD patients [see Table 2; (27–29, 36–41)]. These patients showed high baseline levels of alcohol consumption, were not responders to previous pharmacological treatments, and suffered from other mental or physical disorders. The majority of these patients suffered mainly from anxiety and depression (27, 36, 37), whereas two studies were conducted in AUD patients with clinically-significant liver disease (38, 41). The daily doses of baclofen ranged from 30 to ~150 mg. One study observed that the average dose received by female patients was lower than the one received by males (27). Three studies found that patients without comorbid mental disorders achieved better results compared to patients with comorbid mental disorders [anxiety, depression, and/or borderline personality disorder; 27–29]]. Another study did not find a significant effect of baclofen on either anxiety or depression, which were evaluated using self-reported rating scales (38).

**Table 2:** Observational and retrospective studies.

	**Participants' characteristics**			**Baclofen Treatment**		**Clinical effects of baclofen treatment**		
**References**	**Gender**	**Comorbidity**	**Baseline DDD**	**Mean daily dose**	**Weeks of treatment**	**Alcohol**	**Anxiety**	**Depression**
(27, 36)	70 M 30 F	Other psychiatric disorders (59%) Anxiety disorders (53%) Depression (34%)	M = 19 F = 15	M = 158 mg F = 127 mg	104	↓	*After 3 months, the rate of participants who continued to drink at risk levels was higher among participants with mental disorders (70%) than without (48%)*	
(37)	10 M 3 F	Anxiety (62%) Depression (100%)	~9-33	30-150 mg	Up to 108	↓	Some ↓	-
(38)	31 M 22 F	Liver disease (100%)	20	60 mg	104	↓	↔	↔
(28)	59 M 57 F	Other psychiatric disorders (92%) Anxiety disorders (75%) Depression (56%)	M = ~16 F = ~12	150 mg	52	↓	*After 12 months, the rate of participants with mental disorders was higher among non-abstinent participants (48%) than abstinent participants (12%)*	
(29)	39 M 30 F	23 patients with BPD vs. 46 patients without BPD	At least 8	-	~32	↔ BPD ↓ Controls	*After a serious adverse event, the rate of treatment discontinuation was higher among participants with BPD (65%) than without BPD (6%)*	
(39)	112 M 1 F	Number of patients with comorbid psychiatric illness not provided	–	–	–	↓	–	–
(40)	348 M	No other mental disorders	~12.5	50 mg		↓	–	–
(41)	20 M 15 F	Patients with or without cirrhosis Number of patients with comorbid psychiatric illness not provided	-	30 mg	>23	↓	–	–

*BPD, Borderline Personality Disorder; DDD: Drinks per Drinking Day (1 drink = 12 g of pure alcohol); F: Female; M, Male. Italic indicates rates of participants suffering for the other disorder*.

### Open studies

Table 3 shows open studies in which baclofen was administered to help AUD patients to achieve abstinence or reduce alcohol consumption [see Table 3; (42–47)]. Among these studies, only three provided information on comorbid mental disorders (45–47): in one study (45), five out of twelve participants suffered from mental disorders other than AUD; in the other two studies, patients with severe mental disorders, other than AUD, were excluded (46, 47). In all studies, participants received daily doses of baclofen ranging from 30 to 145 mg and were monitored from 4 to 52 weeks. All these studies reported that baclofen reduced alcohol consumption. Among the studies in which the anxiety and depression levels of participants were evaluated, baclofen administration reduced anxiety but not depression levels (45–47).

**Table 3:** Open-label studies.

	**Participants' characteristics**			**Baclofen treatment**		**Clinical effects of baclofen treatment**		
**References**	**Gender**	**Notes and comorbidities**	**Basal DDD**	**Mean daily dose**	**Weeks**	**Alcohol**	**Anxiety**	**Depression**
(42)	10 M	-	~8	30 mg	4	↓	–	–
(43)	60 N/A	Article in French	-	145 mg	12	↓	–	–
(44)	75 M; 25 F	65 participants suffered from cirrhosis	~7	40 mg	52	↓	–	–
(45)	9 M; 3 F	5 participants suffered from other psychiatric disorders Anxiety levels: BAI = ~5 Depression levels: BDI = ~8	~8	30 mg	12	↓	↓	↔
(46)	80 M (vs. 75 M benfothiamine)	Participants with other psychiatric disorders were excluded Anxiety levels: HAM-A = ~20 Depression levels: HAM-D = 12	-	50 mg	12	↓	↓	↔
(47)	10 M; 6 F	Participants with other psychiatric disorders were excluded Anxiety levels: STAI = ~51 Depression levels: ZUNG = 41	-	30 mg	12	↓	↓	↔

*BAI, Beck Anxiety Inventory (cut off ≥ 10); BDI, Beck's Depression Inventory (cut off > 10); DDD, Drinks per Drinking Day (1 drink = ~12 g of pure alcohol); F, Female; HAM-A, Hamilton Anxiety Rating Scale (cut off > 17); HAM-D, Hamilton Depression Rating Scale (cut off ≥ 8); M, Male; N/A, Not Available; STAI, Spielberger State Trait Anxiety Inventory (cut off ≥ 40); ZUNG, Self-rating depression scale (cut off ≥ 50)*.

### Human laboratory studies

Two laboratory studies investigated the effects of baclofen in non-treatment seeking AUD participants [see Table 4; (26, 48)]. In both studies, participants received 30 mg/day baclofen for approximately a week. In one study, participants with recent (past 6 months) mental disorders, other than AUD, were excluded (48). In the other one, participants had high anxiety levels (see Table 4), but only a few participants had a formal diagnosis, based on DSM-IV, of current anxiety disorders (5 out of 34) or current mood disorder (1 out of 34) (26). One study found that baclofen reduced alcohol self-administration but not cue-elicited craving; furthermore, baseline anxiety levels did not modulate alcohol drinking (48). The other one did not report significant effects of baclofen on either alcohol consumption, cue-elicited craving, or anxiety levels (26). In both studies, baclofen amplified the subjective effects of alcohol, which was suggested as a potential biobehavioral mechanism of how baclofen may affect alcohol drinking (26, 48).

**Table 4:** Human laboratory studies.

	**Participants' characteristics**			**Baclofen Treatment**		**Clinical Effects of Baclofen Treatment**		
**References**	**Gender**	**Notes on comorbidity**	**Baseline DDD**	**Mean daily dose**	**Weeks**	**Alcohol**	**Anxiety**	**Depression**
(26)	14 M; 4 F vs. 13 M; 3 F placebo	Anxiety levels: STAI = ~47	~8	30 mg	1	↔	↔	N/A
(48)	10 M; 4 F	Participants with other psychiatric disorders were excluded	~8	30 mg	1	↔	Baseline anxiety levels did not modulate alcohol drinking	

*DDD, Drinks per Drinking Day (1 drink = 12 g of pure alcohol); F, Female; M, Male;N/A, Not Available; STAI, Spielberger State Trait Anxiety Inventory (cut off ≥ 40)*.

### Randomized double-blind placebo-controlled trials

The findings of the randomized, double-blind placebo-controlled trials of baclofen in the treatment of AUD are outlined in Table 5 (23–25, 49–59). All the studies excluded participants with severe mental disorders other than AUD but, in some studies, participants who received stable doses of antidepressants (24, 55, 56) or participants affected by depression, anxiety, and/or bipolar disorder (51) were allowed to participate. Participants received daily doses of baclofen ranging from 30 to 180 mg for a timeframe ranging from 3 to 26 weeks. All the studies, except two (49, 54), reported the mean values of anxiety and/or depression levels. Most studies used self-reported rating scales such as STAI (Spielberger State Trait Anxiety Inventory) for anxiety and BDI (Beck's Depression Inventory) or ZUNG for depression. Only a few studies used interviewer-rated scales such as HAM-A (Hamilton Anxiety Rating Scale) for anxiety and HAM-D (Hamilton Depression Rating Scale) or MADRS (Montgomery-Asberg Depression Scale) for depression.

**Table 5:** Randomized double-blind placebo-controlled trials.

	**Participants' characteristics**			**Baclofen treatment**		**Clinical effects of baclofen treatment**		
**References**	**Gender**	**Notes and comorbidity**	**DDD**	**Mean daily dose**	**Weeks**	**Alcohol**	**Anxiety**	**Depression**
(23)	BAC: 20 M PLA: 19 M	Participants with severe mental disorders were excluded Anxiety levels: STAI = ~50 Depression levels: ZUNG = ~40	~14	30 mg	4	↓	↓	-
(49)	BAC: 32 M; 10 F PLA: 29 M; 13 F	Participants with severe mental disorders were excluded Cirrhosis	–	30 mg	12	↓	-	-
(50)	BAC: 21 M; 7 F PLA: 11 M; 3 F	Participants with severe mental disorders were excluded Anxiety levels: STAI = ~50 Depression: ZUNG = ~40	~12	30 or 60 mg	12	↓	↔	-
(51)	BAC: 61 M; 28 F PLA: 43 M; 19 F	Participants with severe mental disorders (other than depression, anxiety, and bipolar disorder) were excluded Anxiety levels: STAI = ~50 Depression levels: BDI = ~20	~12	30 or 94 mg	16	↔	↔	↔
(24)	BAC: 22 M; 18 F PLA: 22 M; 18 F	Participants with severe mental disorders (except those with stable doses of antidepressants) were excluded 6 M + 17 F under antidepressants Anxiety levels: STAI = ~40 Depression levels: ZUNG = ~36	~7	30 mg	12	↔	↓	↔
(52)	BAC: 85 M; 3 F PLA: 92 M	Participants with significant psychosis, mania, or elevated risk for suicide were excluded Liver disease Anxiety levels: BSI = 43–52 Depression levels: BDI = 13	~9	30 mg	12	↔	↔	↔
(25)	BAC: 29 N/A PLA: 23 N/A	Participants had a combination of anxiety and depression Anxiety levels: STAI = ~52 Depression levels: ZUNG = ~53	-	37.5 mg	3	-	↓	↓
(53)	BAC: 16 N/A PLA: 16 N/A	Participants with a history of severe mental disorders were excluded Anxiety levels: STAI = ~40 Depression levels: MADRS = ~5.5	-	50 mg	12	↔	↔	↔
(54)	BAC: 9 M; 6 F PLA: 9 M; 6 F	Participants with severe mental disorders were excluded Smoking	–	80 mg	12	↓	–	–
(55)	BAC: 10 M; 18 F PLA: 9 M; 5 F	Participants with severe mental disorders were excluded (except those with stable doses of antidepressants) Anxiety levels: STAI = ~40 41% current anxiety	−16	30 or 60 mg	12	↔↓ in anxious	↔	–
(56)	BAC: 57 M; 20 F PLA: 30 M; 10 F	Participants with severe mental disorders were excluded Liver disease 57 under antidepressants Anxiety levels: DASS anxiety = ~13 Depression levels: DASS depression = ~17	−12.5	30 or 75 mg	12	↓	↔	↔
(57)	BAC: 20 M; 8 F PLA: 19 M; 9 F	Participants with severe mental disorders were excluded Anxiety levels: HAM-A = ~3 Depression levels: HAM-D = ~3		180 mg	12	↓	-	-
(58)	BAC: 24 M; 8 F PLA: 24 M; 8 F	Participants with severe mental disorders were excluded Depression levels: BDI = ~16		50 mg	12	↔	-	↔
(59)	BAC: 118 M; 37 F PLA: 107 M; 48 F	Participants with severe mental disorders were excluded Anxiety levels: HAD Anxiety = ~6 Depression levels: HAD Depression = ~6		153 mg	26	↔	-	-

*BAC, Baclofen; BDI: Beck's Depression Inventory (cut off > 10); BSI, Brief Symptoms Inventory (cut off ≥ 65); DASS, Depression Anxiety Stress Scale; DASS Anxiety (cut off ≥ 8); DASS Depression cut off ≥ 10); DDD, Drinks per Drinking Day (1 drink = 12 g of absolute alcohol); F, Female; HAD, Hospital Anxiety and Depression Scale; HAD Anxiety (cut off ≥ 8); HAD Depression (cut off ≥ 8); HAM-A, Hamilton Anxiety Rating Scale (HAM-A) (cut off >17 mild anxiety); HAM-D, Hamilton Depression Scale (cut off ≥8); M, Male; MADRS, Montgomery-Asberg Depression Scale (cut off ≥ 12); N/A, Not Available; PLA, Placebo; STAI, Spielberger State Trait Anxiety Inventory (cut off ≥ 40); ZUNG (cut off ≥ 50)*.

In two studies, participants had high baseline anxiety levels (23, 50), in two other studies high baseline depression levels (52, 58), and in three studies both high baseline anxiety and depression levels (25, 51, 56). Baclofen administration induced contrasting results regarding alcohol consumption. It reduced alcohol consumption in some studies (23, 49, 50, 54, 55, 57), but not in others (24, 51–53, 55, 58, 59). One study suggested a relationship between comorbid anxiety and treatment response to baclofen (55). In this study, baclofen administration reduced alcohol consumption in anxious patients, but did not induce significant modifications in other participants. However, this relationship was not observed in other studies. Baclofen reduced alcohol consumption in studies in which participants had high (23, 50, 56) or low (57) baseline anxiety levels. On the other hand, baclofen failed to modify alcohol consumption in other studies in which participants had high (51) or low (59) baseline anxiety levels. No study provided the results obtained specifically in participants affected by other mental disorders.

## Discussion

The presence of another mental disorder, such as anxiety and depression, other than AUD represents a frequent and complex clinical phenomenon (1, 14, 60). The pharmacological treatment of AUD patients affected by comorbid mental disorders represents a significant challenge (61, 62). AUD and a second mental disorder may occur independently, or one of the two disorders may have influenced the development of the other one (1, 63). For instance, an anxious patient may develop AUD as consequence of the excessive alcohol consumption to self-medicate anxiety. On the other hand, AUD patients frequently develop temporary alcohol-induced depressive and/or anxiety symptoms during intoxication and/or withdrawal (63). Accordingly, physicians need to establish if AUD patients with a comorbid mental disorder require a pharmacological treatment for both the conditions (e.g., if both the conditions are severe and long lasting) or not (e.g., if co-occurrent symptoms are alcohol-induced and tend to resolve without treatment within 3–4 weeks) (63).

According to preclinical studies, GABA_B_ receptors modulate anxiety and depression-related behaviors (21), and baclofen may induce better results among AUD patients affected by anxiety and/or mood disorders through a reduction of the severity of these disorders. The present narrative mini-review was aimed at investigating the role of psychiatric comorbidity in explaining potential different responses to baclofen treatment among AUD patients. The results show that the majority of AUD patients treated with baclofen and described by the case reports, as well as the observational and retrospective studies, suffered from anxiety and/or mood disorders. This finding agrees with data of large epidemiological studies (14, 60). However, the results of the present review also show that, despite these disorders being common among AUD patients, patients with severe mental disorders (including severe anxiety and mood disorders) were excluded by the randomized, controlled, trials (RCTs) conducted to evaluate the efficacy of baclofen to treat AUD. Therefore, these results do not allow us o evaluate whether baclofen efficacy is different in AUD patients with comorbid psychiatric disorders compared to those without. One RCT found that baclofen administration reduced alcohol consumption in anxious patients, but not in patients with low levels of anxiety (55). However, in this study, baclofen administration did not significantly reduce anxiety levels. A recent meta-analysis also found no difference between baclofen and placebo in reducing both depression and anxiety levels among AUD patients (64). The results of the present review may be useful to better understand these results. Indeed, both these studies (55, 64) evaluated the efficacy of baclofen in modifying the severity of anxiety or depression among AUD patients not affected by severe mental disorders. In addition, the RCTs used different scales to measure the severity of anxiety and/or depression, such as self-reported rating scales (e.g., STAI, BDI, and ZUNG) and interviewer-rated scales (e.g., HAM-A, HAM-D, and MADRS). The exclusion of patients with severe mental disorders, and the variability in the scales adopted, prevents current meta-analyses to evaluate potential differences in baclofen efficacy in reducing the severity of the comorbid mental disorders. To reduce the variability, the interviewer-rated scales should be used rather than self-reported rating scales (65, 66). Accordingly, it is desirable that future RCTs investigate the efficacy of baclofen in AUD patients affected by other mental disorders, using interviewer-rated scales to establish the effects on the severity of anxiety and/or depression.

## Conclusions

There is a critical need to develop novel medications for AUD. In addition, the new diagnostic criteria of AUD in the DSM-5 may increase the prevalence of new cases of this mental disorder, compared to the criteria provided by the DSM-IV (67), therefore the importance of developing new effective treatments is even greater. The GABA_B_ receptor represents a promising pharmacological target, a concept that has been supported by a plethora of animal studies. Medication development for CNS indications is challenging and, indeed, the translation of findings from animal models to humans is one of the most difficult steps in this field. The presence of both positive and negative RCTs is not uncommon in the AUD literature. The medication development field, at large, and the results of recent meta-analyses show baclofen is not exempt from this pattern (65, 68, 69). Inconsistencies across studies may be due to several factors, which may include (but are not limited to) differences at several levels, e.g., (a) medication-related differences like baclofen doses, medication adherence, interactions with other concomitant medications, or pharmacokinetics; (b) patient-related differences like gender, severity of AUD, tolerance to baclofen's effects, psychiatric comorbidities, or medical comorbidities; and (c) geographic- and site-related differences like specific patient-provider interactions, or amount and type of behavioral interventions provided in addition to the medication. These factors are likely to explain not only why RCTs have yielded different results, but also why analyses focused on one specific factor (e.g., baclofen dose, patients with anxiety comorbidity) have also generated conflicting results. Despite the lack of consistent evidence of efficacy, baclofen is frequently used off-label to treat AUD, especially in some European countries and Australia (70). It is preferable that future prospective studies and meta-analytical efforts try to look at the interactions of all these factors as they relate to the medication, patient, and site, in order to identify which are, if any, the best variables that may predict the patient's phenotype that is most likely to benefit from the treatment with baclofen. Finally, it is important to keep in mind that, while baclofen represents the prototypic agonist of the GABA_B_ receptor, very promising rodent data suggest the GABA_B_ positive allosteric modulators may represent a better pharmacological approach (19). Therefore, future efforts should also focus on profiling these compounds from a toxicology and safety standpoint, in order to bring them to the clinical setting and test their safety and potential efficacy in humans.

## Author contributions

RA led the literature search and drafted the manuscript. LL provided substantial contributions to the intellectual content of the manuscript. Both authors approved the final version of the manuscript.

### Conflict of interest statement

The authors declare that the research was conducted in the absence of any commercial or financial relationships that could be construed as a potential conflict of interest.
